# When Winters Fail: How Warming Winters Fuel Pest Resurgence and Threaten Food Security

**DOI:** 10.1002/pei3.70216

**Published:** 2026-09-24

**Authors:** Mostafa Farajpour, Shahab Mirinejad, Mitra Moezipour, Mohammad Sadat‐Hosseini

**Affiliations:** ^1^ Mazandaran Agricultural and Natural Resources Research and Education Center Agricultural Research, Education and Extension Organization (AREEO) Sari Iran; ^2^ Department of Horticultural Science, Faculty of Agriculture University of Jiroft Jiroft Iran

**Keywords:** *Ceratitis capitata*, climate change, food security, integrated pest management, invasive species, organophosphate, thermal tolerance

## Abstract

The Mediterranean fruit fly ( *Ceratitis capitata* , Wiedemann; Diptera: Tephritidae), commonly known as the Medfly, re‐emerged in Mazandaran province, northern Iran—home to an estimated 25% of the nation's fruit crops and approximately 80% of its citrus production—following its apparent eradication in 1984, an outcome consistent with several years of pheromone‐trap surveillance without further captures. Its return in 2006 and subsequent spread is consistent with a broader phenomenon we term the erosion of climatic pest barriers: seasonal thermal conditions that have historically suppressed or excluded pest populations, and which appear to be weakening as winters warm. Warming may facilitate Medfly persistence by relaxing overwintering limitation, although reinvasion pressure, host availability, fruit movement, and surveillance and management practices likely also contribute. In Mazandaran, growers report substantial on‐tree citrus losses during heavy infestation years, and pest‐induced fruit wounding may provide infection courts for common postharvest fungal pathogens, compounding losses for smallholder producers. Current management relies heavily on organophosphate insecticides, particularly malathion, whose hazard classification and associated risks are well documented, even though realized exposure‐related risk in practice depends on application conditions. We situate the Mazandaran case within a broader global pattern of climate‐associated pest range expansion and weakened biological control, and argue that resilience‐based alternatives—including the Sterile Insect Technique, entomopathogenic agents, and threshold‐guided digital monitoring, embedded within climate‐smart agriculture frameworks—offer a technically proven, though not yet regionally established, pathway forward. We propose that climatic pest barriers merit recognition as an ecosystem service whose erosion under warming carries implications for temperate agricultural systems beyond this case.

## 1 Introduction

Climate change is reconfiguring the geography of agricultural risk. As global mean temperatures rise and winter cold events become shorter and less severe, the thermal boundaries that have historically limited many invasive pest species from establishing in temperate agricultural zones may be eroding. Among the most economically consequential beneficiaries of this shift is the Mediterranean fruit fly ( *Ceratitis capitata* , Wiedemann), a polyphagous pest capable of infesting more than 300 host plant species and considered one of the most destructive agricultural insects globally (Giunti et al. 2023). Its resurgence in Mazandaran province, northern Iran, after decades of apparent eradication illustrates a challenge that may increasingly confront agricultural systems at the warm margins of temperate zones worldwide.

The central argument of this Insight is that warming winters are eroding what we term *climatic pest barriers*: seasonal thermal conditions—related to, but distinct from, overwintering limitation, thermal niche boundaries, seasonal mortality, and climatic suitability more broadly—that have historically suppressed or excluded pest populations on a recurring seasonal basis. We propose this term as a conceptual framing for the present manuscript, intended to draw attention to seasonal cold mortality as a natural regulatory service that is rarely recognized until it fails. The Mazandaran Medfly case is presented here as an illustrative example of this broader phenomenon, rather than as direct proof that warming alone has driven the pest's resurgence.

Mazandaran's significance extends well beyond its geography. The province contributes approximately 25% of Iran's total fruit production and roughly 80% of its citrus output (Mirsardoo et al. 2010). Its re‐infestation by Medfly—apparently eradicated there following extreme winter cold in 1984 and redetected in 2006—is consistent with the erosion of climatic pest barriers under a documented regional warming trend, although reinvasion pressure, produce movement, and changes in surveillance or management practices may also have contributed. Where such barriers weaken, the consequences may cascade from the orchard to the warehouse, from smallholder income to regional food security, and from targeted pesticide use to broader occupational and environmental exposure.

This Interaction Insight describes the Medfly resurgence in Mazandaran as an illustrative plant–environment interaction: one in which a warming climate may be restructuring the ecological relationship between a crop system and an invasive herbivorous pest. We examine the evidence for range expansion, the plausible cascading impacts on smallholder livelihoods and food security, the limitations of current chemical‐centred responses, and the evidence base for more sustainable alternatives. We situate this Iranian case within global patterns—from California quarantine risk to European range expansion—to indicate that the lessons from Mazandaran may carry relevance for food systems elsewhere, while recognizing that direct extrapolation requires caution.

## 2 The Medfly in Mazandaran: A Climate‐Associated Resurgence

**FIGURE 1:**
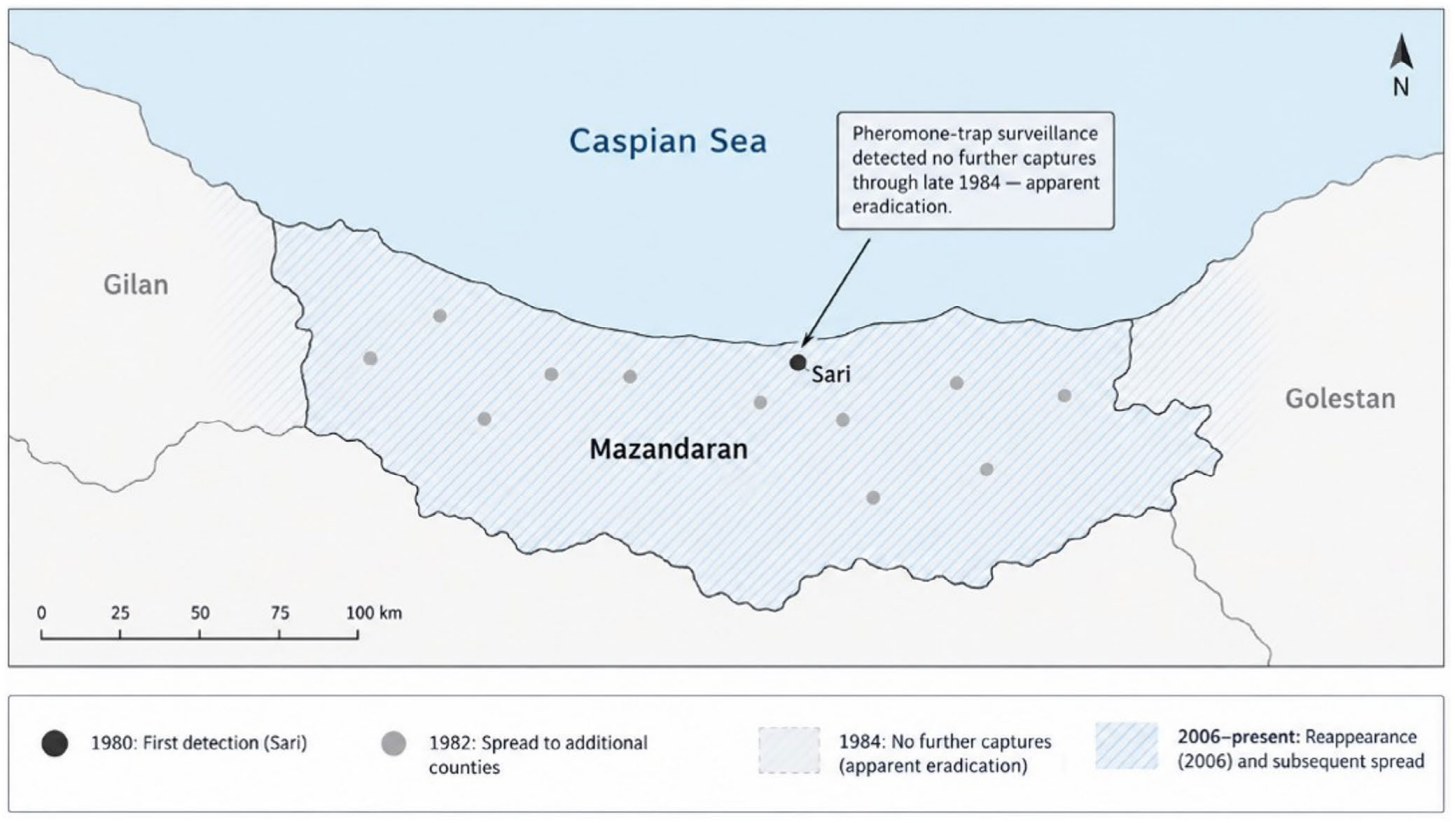
Schematic reconstruction of the documented spatiotemporal spread of *Ceratitis capitata* in Mazandaran province, northern Iran, based on detection records reported in Mirsardoo et al. (2010).

*Ceratitis capitata* was first recorded in Mazandaran province (Sari) in 1980, and by 1982 had been collected in other counties on additional hosts, with the heaviest infestation occurring in orchards adjacent to residential areas. Continued pheromone‐trap surveillance in the years that followed detected no further captures through late 1984, consistent with successful, if not exhaustively documented, eradication—likely aided by the region's cold winters (Mirsardoo et al. 2010). Its reappearance in 2006 coincided with a documented regional warming trend, and populations have since expanded across Mazandaran and into adjacent provinces (Figure 1). This pattern—apparent local eradication followed by climate‐associated re‐invasion—is consistent with, though not sole proof of, how rising temperatures may restructure the invasion landscape.

The thermal biology of *C. capitata* is central to understanding its resurgence, though several related but distinct concepts are worth separating. Critical thermal minima describe the temperature at which physiological function fails; cold tolerance describes an organism's capacity to survive short‐term cold exposure; overwintering capacity describes survival across an entire cold season; and permanent establishment requires that a population persist and reproduce across multiple years without renewed introduction (Table 1). Adults of *C. capitata* experience critical thermal minima of approximately 5.4°C–6.6°C, and larval and pupal stages show differential cold tolerance across geographically distinct populations (Papadopoulos et al. 2023). Recent laboratory and field investigations indicate that Medfly is capable of overwintering—though not yet demonstrated to permanently establish—in urban and peri‐urban environments at latitudes as high as 48° N in central Europe, particularly where microhabitats buffer populations against extreme temperatures (Papadogiorgou et al. 2024). Climate suitability modeling, incorporating CLIMEX projections and historical trapping records, indicates that the potential geographical range of *C. capitata* has expanded substantially since 1970, with the rate of expansion accelerating from the 1990s onward (Szyniszewska et al. 2024).

**TABLE 1:** Comparative thermal biology of *Ceratitis capitata* across studied populations.

Parameter	Reported value	Population/context	Source
Critical thermal minimum (adults)	~5.4°C–6.6°C	Mixed populations	Papadopoulos et al. (2023)
Upper thermal limit	~42°C–44°C	Geographically distinct populations	Papadopoulos et al. (2023); Szyniszewska et al. (2024)
Documented overwintering latitude	Up to 48° N	Central Europe (urban/peri‐urban microhabitats)	Papadogiorgou et al. (2024)
Range expansion trend	Accelerating from 1990s onward	Global (CLIMEX modeling)	Szyniszewska et al. (2024)

**FIGURE 2:**
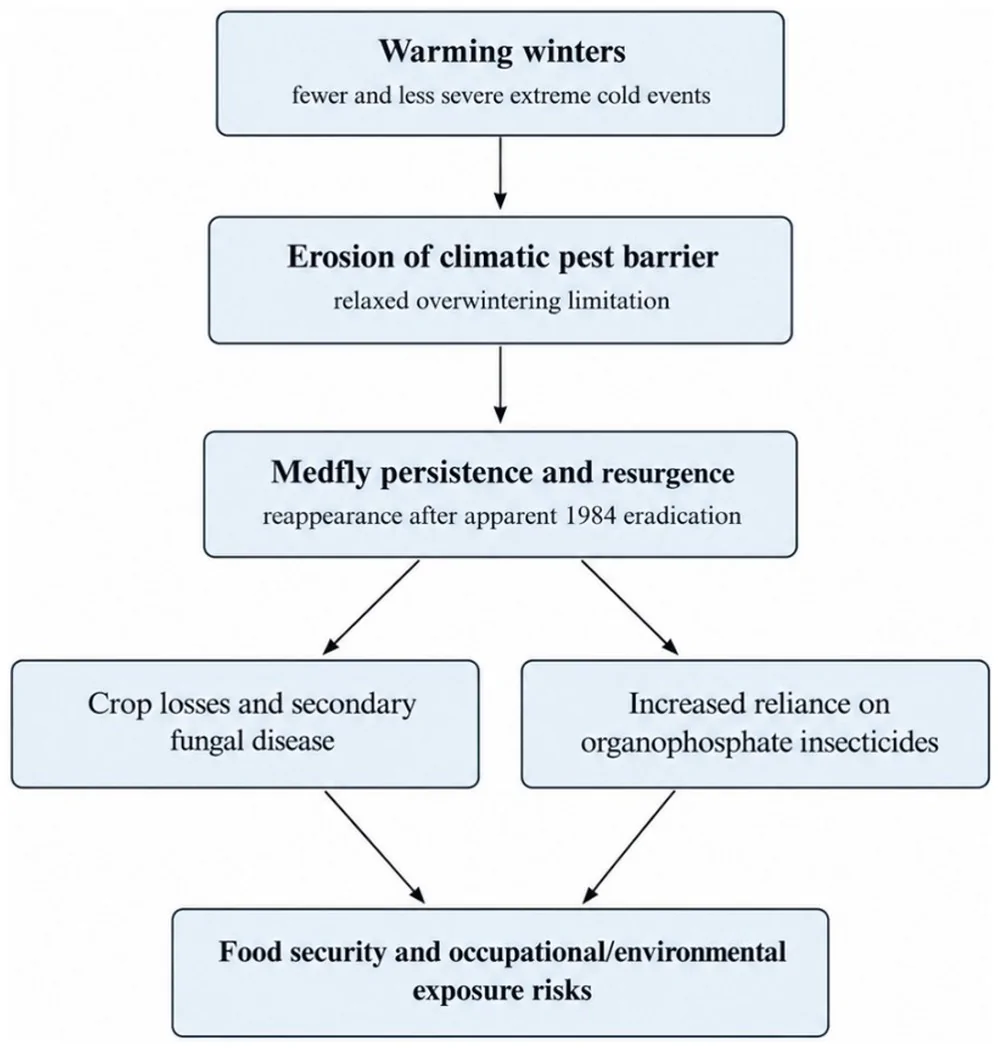
Conceptual flow diagram illustrating the hypothesized relationship between warming winters, the erosion of climatic pest barriers, and downstream impacts on crop production, pesticide dependency, food security, and human and environmental exposure in Mazandaran province.

In Mazandaran, growers and regional agricultural specialists report substantial on‐tree citrus losses during heavy infestation years, with figures in the range of 40%–50% cited in local accounts, as larvae tunnel through fruit flesh and render produce unmarketable. To our knowledge, these figures have not been derived from systematic regional surveys and should be interpreted as indicative local estimates rather than precise measurements. More critically, pest‐induced fruit wounding may create entry points for secondary fungal pathogens—chiefly *Penicillium digitatum*, *P. italicum* , and *Geotrichum candidum*—which are established causes of green mold, blue mold, and sour rot in citrus more generally (Rovetto et al. 2024); Medfly‐associated wounds may provide infection courts for these pathogens where damage co‐occurs with warehouse storage, compounding post‐harvest losses. The interaction between primary pest damage and secondary pathogen invasion represents a plant–environment–pathogen cascade that conventional single‐organism management frameworks are poorly equipped to address, although direct local evidence linking Medfly injury specifically to these named pathogens in Mazandaran has not, to our knowledge, been systematically documented. The proposed pathway linking warming, the erosion of climatic pest barriers, and downstream food‐security impacts is summarized conceptually in Figure 2.

Elevated summer temperatures may further accelerate Medfly population dynamics. Warmer conditions can shorten generation time, increase the number of reproductive cycles per season, and may be selecting for populations with enhanced heat resistance, as suggested by plasticity studies showing geographic variation in upper thermal limits ranging from approximately 42°C to 44°C (Papadopoulos et al. 2023; Szyniszewska et al. 2024). The compounding effects of milder winters—potentially enabling survival—and hotter summers—potentially accelerating reproduction—may create demographic pressure that outpaces the management capacity of smallholder farmers operating with limited resources.

## 3 A Global Pattern: Warming and the Erosion of Climatic Pest Barriers

The Mazandaran case is unusual in its documentation, but it is illustrative of a broader phenomenon. Deutsch et al. (2018) projected that, per degree of global mean surface warming, insect‐driven yield losses in wheat, rice, and maize could increase by a further 10%–25%; a more recent synthesis (Ma et al. 2025) projects additional yield losses of 46%, 19%, and 31% for these crops respectively under 2°C warming, and finds that pests at mid‐ to high‐latitudes—precisely those regions where cold winters have historically served as a control mechanism—show the strongest positive responses to warming, through expanded geographic ranges, advanced phenology, and additional reproductive generations annually.

Two further cases illustrate specific mechanisms relevant to the Medfly case. In North America, the fungal entomopathogen *Entomophaga maimaiga*, which has historically provided biological control of the invasive spongy moth ( *Lymantria dispar* ), is projected to become less effective under warming and drying conditions—illustrating how climate change can weaken an existing biological control service (Liu et al. 2025). In sub‐Saharan Africa, the fall armyworm ( *Spodoptera frugiperda* ) has spread across more than 44 countries, a pattern partly enabled by climatic conditions permitting year‐round reproduction and rapid range expansion—illustrating climate‐associated range expansion in a pest of major economic importance (Haider et al. 2026). Both cases follow a broadly similar logic to the Mazandaran example: warming may weaken the thermal or biological mechanisms that historically constrained pest populations, creating conditions that existing management systems were not designed to handle—though the specific pathways differ across systems and warrant caution in direct comparison.

The wider economic stakes of Medfly range expansion are considerable, if illustrative rather than directly transferable: suitability models suggest that quarantine measures triggered by Medfly establishment in California could cost an estimated 132,000 jobs and $13.4 billion in economic losses (Hoy 2019), indicating the scale of impact possible from range expansion into new production regions, though this projection concerns a different geographic and regulatory context from Mazandaran. In Iran more broadly, climate change has been linked to declining agricultural productivity across multiple commodity classes; the pistachio sector, a globally significant export crop, has been described as a documented example of climate‐driven systemic stress (Farajpour 2025). Iran's cultivated agricultural land has been reported to have declined in recent decades, with water scarcity, land degradation, and soil erosion described as substantially exceeding regional averages (Iran Daily 2025); we note that this specific figure derives from a media report rather than a peer‐reviewed or official statistical source, and that confirmation against government or FAO datasets would strengthen it. These structural vulnerabilities may amplify the impact of climate‐associated pest resurgence on food security, though the connection to the Medfly case specifically remains inferential.

Globally, a substantial share of crop production is lost annually to pests and diseases, and climate change is projected to worsen this trajectory (Tang et al. 2023). These losses are widely thought to fall disproportionately on smallholder farming systems in lower‐income regions, which tend to have the least adaptive capacity and are most dependent on natural regulatory services—including cold‐season pest mortality—that a warming climate may be eroding. Table 2 summarizes these local and comparative dimensions of the Medfly resurgence.

**TABLE 2:** Summary of key dimensions of the Medfly resurgence in Mazandaran, distinguishing local observations from comparative evidence drawn from elsewhere.

Dimension	Observation	Evidence basis	Implication	Key references
Pest resurgence	Medfly re‐emerged in Mazandaran in 2006; reported absent following 1984 eradication	Local (Mazandaran)	Warming winters may allow re‐establishment where climatic barriers weaken	Mirsardoo et al. (2010); Szyniszewska et al. (2024)
Crop losses	On‐tree citrus loss of 40%–50%; postharvest fungal losses reported up to 70% (local, media‐reported estimates)	Local (Mazandaran, not systematically surveyed)	Substantial direct economic impact on smallholder producers, though unverified against systematic data	—
Fungal disease following injury	Medfly‐associated wounds may serve as infection courts for common postharvest fungal pathogens (e.g., *Penicillium* spp., *Geotrichum candidum*)	External (comparative, citrus pathology literature)	Pest injury may increase susceptibility to secondary fungal infection, compounding losses	Rovetto et al. (2024)
Thermal biology	Medfly overwintering demonstrated at latitudes up to 48° N in Europe	External (comparative)	Temperate zones previously buffered by cold may be becoming climatically suitable	Papadogiorgou et al. (2024); Papadopoulos et al. (2023)
Global range risk	Potential quarantine in California fruit estimated at $13.4 bn, 132,000 jobs	External (comparative)	Illustrates scale of possible economic risk from range expansion elsewhere	Hoy (2019); Ma et al. (2025)
Insecticide burden	Malathion (organophosphate) dominates control in Iran; WHO high‐hazard classification	Local practice; hazard classification is external	Heavy reliance carries hazard‐ and exposure‐related risks	Jepson et al. (2020); Giunti et al. (2023)
Sustainable IPM	SIT + parasitoid release achieved ~98% suppression in Mexico	External (comparative)	Demonstrates technical potential; regional feasibility in Iran not yet established	Cancino et al. (2023)
Threshold‐based spraying	Threshold‐guided programmes cut insecticide use 44%, costs 40% (global meta‐analysis)	External (comparative)	Knowledge‐based frameworks may outperform blanket chemical use	Leach et al. (2025)

## 4 Organophosphate Dependency: Hazard, Exposure, and Diminishing Returns

The dominant management response to Medfly in Mazandaran—as in many affected regions—is the protein‐baited application of organophosphate (OP) insecticides, principally malathion. Malathion is classified by the World Health Organization as a high‐hazard pesticide requiring maximum personal protective equipment and engineering and behavioral mitigations for safe use (Jepson et al. 2020); this hazard classification describes the inherent toxicological properties of the compound rather than the level of risk under any particular set of field conditions. The realized, exposure‐related risk instead depends on how the pesticide is applied: unmonitored, frequent, and often unprotected applications—as commonly reported in Mazandaran and comparable settings—raise the likelihood of cumulative exposures approaching or exceeding safety thresholds, particularly for agricultural workers, their families, and children, who may be disproportionately vulnerable to OP‐associated neurotoxicity.

The compound's primary mechanism—inhibition of acetylcholinesterase—underlies a spectrum of reported health effects ranging from acute neurotoxic symptoms to chronic neurological impairment; the International Agency for Research on Cancer has classified malathion as a probable human carcinogen (Giunti et al. 2023). Beyond human health, repeated spraying disrupts beneficial insect communities, including pollinators and natural enemies of secondary pests, which can generate further pest outbreaks requiring additional applications.

The central concern raised here is not primarily toxicological but relates to dependency: climate‐driven pest persistence appears likely to intensify reliance on chemical control in the absence of alternatives, compounding environmental and occupational exposure over time and potentially accelerating resistance. This dependency is also difficult to justify on management grounds alone. A global meta‐analysis found that threshold‐based insecticide programmes—in which applications are triggered by pest population monitoring rather than calendar schedules—reduced insecticide use by 44% and costs by 40% without compromising yield, while also increasing beneficial insect abundance (Leach et al. 2025). This evidence suggests that blanket chemical approaches in Mazandaran may be both costlier and less effective than knowledge‐based alternatives, independent of their toxicological costs.

## 5 Pathways to Resilience: Integrated Pest Management and Climate‐Smart Agriculture

A transition away from chemical dependency toward more sustainable Medfly management would likely require the integration of multiple complementary strategies, deployed at the landscape scale and embedded in supportive policy and financing frameworks. We present the following approaches as evidence of technical potential rather than as proven solutions for northern Iran specifically, since none has yet been implemented at scale in this context.

### 5.1 Sterile Insect Technique and Biological Control

Sterile Insect Technique (SIT)—the mass rearing, sterilization, and release of male insects to compete with wild populations—is among the most operationally mature non‐chemical Medfly suppression methods. In controlled trials combining SIT with augmentative releases of the parasitoid *Diachasmimorpha longicaudata* in mango‐producing areas of Mexico, population suppression of approximately 98% was achieved (Cancino et al. 2023); this result was obtained within an established, well‐resourced regional programme with mature mass‐rearing and release infrastructure, and comparable suppression under the smallholder‐dominated production conditions of northern Iran—where such infrastructure does not yet exist—has not been demonstrated. Modeling of re‐mating and residual fertility indicates that SIT efficacy is sensitive to operational parameters, and that optimized programmes accounting for these dynamics can achieve substantially greater suppression than naive mass‐release approaches (Dumont and Oliva 2024). Establishing comparable SIT infrastructure in Iran may be technically feasible but would require sustained investment in mass‐rearing capacity, area‐wide coordination, and regulatory support.

### 5.2 Entomopathogenic Agents

Entomopathogenic nematodes and fungi offer additional biological control tools that could be integrated with SIT to target soil‐dwelling life stages. These approaches remain underutilized in Iranian Medfly management but have demonstrated efficacy in other pest systems.

### 5.3 Precision Monitoring and Digital Agriculture

Real‐time monitoring using pheromone traps, combined with microclimate data and predictive modeling, could in principle enable threshold‐based spray decisions that reduce unnecessary pesticide use. Remote sensing and machine‐learning tools are increasingly used for early pest detection and population forecasting at landscape scales (Woźniak and Ijaz 2024; Ahmed et al. 2024). Realizing this potential in smallholder systems such as those in Mazandaran would depend on overcoming practical constraints, including rural connectivity, trap‐network maintenance and coverage, and local validation of remotely sensed or modeled predictions against ground conditions.

### 5.4 Climate‐Smart Agriculture

Pest management measures alone are unlikely to be sufficient without a broader enabling environment. Climate‐Smart Agriculture (CSA) frameworks that couple adaptive pest management with crop insurance, agro‐advisory services, and targeted financing have been associated with improved food security and income outcomes for smallholders elsewhere (Lipper et al. 2014; Begna and Wakweya 2025; Ewulo et al. 2025). In Iran, equitable policy design would additionally be important to ensure that climate mitigation instruments do not transfer costs to smallholder farmers already affected by Medfly losses.

Together, these approaches point toward a resilience‐based rather than a purely reactive management paradigm, sustaining ecosystem services—predation, parasitism, and natural thermal regulation—that have historically kept pest populations at tolerable levels, while acknowledging that translating this potential into practice in northern Iran will require investment and institutional capacity not yet in place.

## 6 Uncertainty and Alternative Explanations

The interpretation offered here—that warming winters have facilitated Medfly persistence in Mazandaran by eroding a climatic pest barrier—is consistent with the regional evidence but is not the only possible explanation for the pest's resurgence and spread. Increased movement of infested fruit and planting material, growth in host availability through orchard expansion, reinvasion pressure from neighboring regions, changes in surveillance intensity, and shifts in pest‐management practices since the 1980s could each have contributed independently or in combination with climatic change. We regard warming as a plausible and important facilitating factor rather than a demonstrated sole cause, and note that disentangling these contributions would require dedicated empirical research—for example, long‐term trapping records analyzed alongside meteorological and land‐use data—that lies beyond the scope of this Insight.

## 7 Conclusions and Forward Look

The reappearance and spread of *Ceratitis capitata* in Mazandaran province is, in our view, best understood not primarily as a story about a single insect pest, but as an illustration of how a climatic regulatory service—winter cold—that agricultural systems have long depended upon, often without recognizing its value, may be eroding under warming. Where this service weakens, the consequences may extend through crop systems, food supply chains, smallholder livelihoods, and rural health in ways that conventional, reactive pest management is poorly positioned to address.

We draw three tentative conclusions with potential relevance beyond this case. First, climatic pest barriers may be usefully thought of as an ecosystem service whose value becomes apparent mainly in its absence, and their mapping and consideration could be integrated into climate adaptation planning alongside more widely recognized services such as pollination. Second, chemical‐centred management carries costs—health risks for vulnerable rural communities, resistance, and secondary pest outbreaks—that appear to work against its own stated aims. Third, technically proven alternatives such as SIT, biological control, and threshold‐based management exist, but translating them into practice in smallholder‐dominated systems such as Mazandaran's will require investment, institutional capacity, and policy support that these systems cannot generate on their own.

As winters continue to warm, the situation observed in Mazandaran may become more common elsewhere. Whether the institutional, financial, and scientific capacity exists to respond before the cumulative costs to food systems and rural health become substantial is, we suggest, the central open question this case raises.

## Funding

The authors have nothing to report.

## Conflicts of Interest

The authors declare no conflicts of interest.

## Data Availability

Data sharing not applicable to this article as no datasets were generated or analyzed during the current study.
